# Enhanced Visualization of Subtle Outer Retinal Pathology by *En Face* Optical Coherence Tomography and Correlation with Multi-Modal Imaging

**DOI:** 10.1371/journal.pone.0168275

**Published:** 2016-12-13

**Authors:** Danuta M. Sampson, David Alonso-Caneiro, Avenell L. Chew, Tina Lamey, Terri McLaren, John De Roach, Fred K. Chen

**Affiliations:** 1 Centre for Ophthalmology and Visual Science, The University of Western Australia, Crawley, Western Australia, Australia; 2 Lions Eye Institute, Nedlands, Western Australia, Australia; 3 Contact Lens and Visual Optics Laboratory, School of Optometry and Vision Science, Queensland University of Technology, Brisbane, Queensland, Australia; 4 Department of Medical Technology and Physics, Sir Charles Gairdner Hospital, Hospital Ave, Nedlands, Western Australia, Australia; 5 Department of Ophthalmology, Royal Perth Hospital, Perth, Western Australia, Australia; University of Florida, UNITED STATES

## Abstract

**Purpose:**

To present *en face* optical coherence tomography (OCT) images generated by graph-search theory algorithm-based custom software and examine correlation with other imaging modalities.

**Methods:**

*En face* OCT images derived from high density OCT volumetric scans of 3 healthy subjects and 4 patients using a custom algorithm (graph-search theory) and commercial software (Heidelberg Eye Explorer software (Heidelberg Engineering)) were compared and correlated with near infrared reflectance, fundus autofluorescence, adaptive optics flood-illumination ophthalmoscopy (AO-FIO) and microperimetry.

**Results:**

Commercial software was unable to generate accurate *en face* OCT images in eyes with retinal pigment epithelium (RPE) pathology due to segmentation error at the level of Bruch’s membrane (BM). Accurate segmentation of the basal RPE and BM was achieved using custom software. The *en face* OCT images from eyes with isolated interdigitation or ellipsoid zone pathology were of similar quality between custom software and Heidelberg Eye Explorer software in the absence of any other significant outer retinal pathology. *En face* OCT images demonstrated angioid streaks, lesions of acute macular neuroretinopathy, hydroxychloroquine toxicity and Bietti crystalline deposits that correlated with other imaging modalities.

**Conclusions:**

Graph-search theory algorithm helps to overcome the limitations of outer retinal segmentation inaccuracies in commercial software. *En face* OCT images can provide detailed topography of the reflectivity within a specific layer of the retina which correlates with other forms of fundus imaging. Our results highlight the need for standardization of image reflectivity to facilitate quantification of *en face* OCT images and longitudinal analysis.

## Introduction

Diseases of the human retina can affect a single layer of this multi-layered semi-transparent tissue. The varying impact of diseased retinal tissue on absorption, scattering, fluorescence and reflection of light as seen *en face* through bio-microscopy provides important clues in differential diagnosis of retinal diseases. Capitalizing on these optical properties, multimodal retinal imaging has evolved into the standard of care [1].

The most common retinal imaging modalities are: color fundus photography, near-infrared reflectance (NIR), fundus autofluorescence (FAF), fluorescence angiography (FA) and scanning-laser ophthalmoscopy (SLO) [1]. However, these modalities can only provide two-dimensional *en face* images which cannot reveal subtle alteration within a single layer of the retina [2]. In the last 2 decades, optical coherence tomography (OCT) has become an indispensable retinal imaging modality because it captures a three-dimensional image of the retina [3]. However, direct comparison between the *en face* images obtained from NIR, FAF, FA, SLO and the cross-sectional OCT images is not possible because these imaging planes are orthogonal [4]. Such limitation can be overcome by alignment of consecutive OCT scans, volume reconstruction and retinal layer segmentation to generate an *en face* image of each layer of the retina [5–18]. Various terminologies have been used to describe this method of OCT images visualization, including C-scan [4], OCT fundus image [11], projection OCT fundus [14] and *en face* OCT [19]. In this paper, the term “*en face* OCT” will be used to encompass all these concepts. The increased scattering and reflection of the infrared light from OCT at the level of the retinal nerve fiber layer, inner and outer plexiform layers and the four outer retinal bands (external limiting membrane, ellipsoid zone, interdigitation zone and retina pigment epithelium-Bruch’s membrane complex) have been used to generate *en face* OCT images to detect subclinical disease. *En face* OCT images have been correlated with fundus photography [10,14,20–22], SLO [4], fluorescein angiography [10,14], fundus autofluorescence [19,23] and functional measures [19,24]. However, the ability to reconstruct *en face* view from OCT scans in diseased retina is limited by frequent segmentation error in the identification of the retinal pigment epithelium and Bruch’s membrane. Furthermore, correlation between lesions seen in *en face* OCT image with pathology seen on multimodal imaging is lacking.

Herein we describe a custom software that incorporates a graph-search theory based algorithm to generate *en face* OCT images of the outer retinal layers from OCT B-scans acquired from a commercial spectral domain (SD) OCT device. We determine the optimal image acquisition protocol for generating *en face* OCT images of adequate quality and compare the segmentation results between the custom software and commercial software. Finally, we use four unique retinal diseases to illustrate the utility of *en face* OCT image by correlating with SLO-derived reflectance (NIR), autofluorescence (infrared autofluorescence, IRAF; blue-light autofluorescence, BAF), microperimetry and adaptive optics flood-illumination ophthalmoscopy (AO-FIO).

## Materials and Methods

This study was approved by The University of Western Australia Human Ethics Research Office (RA/4/1/7916) and written informed consent was obtained from all subjects.

### Subjects

Retinal images of subjects prospectively enrolled into the Western Australian Retinal Degeneration Study were used for analysis. Images from three healthy controls were chosen for optimization of OCT image acquisition protocol in order to maximize *en face* image quality with minimum raster density. Retinal images from four patients with various types of retinal diseases were selected to illustrate the utility of *en face* OCT reconstruction in four unique clinical scenarios (Table 1): (1) clearly visible lesion on SLO but no obvious lesion on OCT B-scans (angioid streaks in pseudoxanthoma elasticum), (2) subtle lesions on both SLO and OCT B-scans (acute macular neuroretinopathy), (3) no obvious lesions on either SLO or OCT B-scans (early hydroxychloroquine toxicity) and (4) no obvious lesion on SLO but clearly visible lesion on OCT B-scans (Bietti crystalline dystrophy).

**Table 1:** Description of illustrative cases.

Cases	SLO lesion visibility	AO-FIO lesions visibility	OCT B-scan lesion visible	*En face* OCT image lesions visibility	Diagnosis
1	Yes	Yes	No	Clearly seen	ABCC6
	(angioid streak)	(angioid streak)	(Normal RPE layer)	(angioid streak)	
2	Subtle	Yes	Subtle	Clearly seen	AMN
	(dark patch)	(cone loss)	(Loss of EZ, IZ)	(zone of EZ/IZ loss)	
3	No	Yes	No	Clearly seen	HCQ
	(no lesion seen)	(cone loss)	(Attenuated IZ only)	(zone of IZ loss)	
4	No (diffuse	Yes	Yes	Clearly seen	CYP4V2
	RPE loss)	(preserved cone)	(Preserved EZ, RPE)	(zone of RPE)	

SLO: scanning laser ophthalmoscopy; AO-FIO: adaptive optics flood illumination ophthalmoscopy; OCT: optical coherence tomography; RPE: retinal pigment epithelium; EZ–ellipsoid zone; IZ–interdigitation zone; ABCC6: ATP-binding cassette sub-family C member 6 (pseudoxanthoma elasticum); AMN: acute macular neuroretinopathy; HCQ: hydroxychloroquine; CYP4V2: cytochrome P450 family 4 subfamily V polypeptide 2 (Bietti crystalline dystrophy)

### Clinical assessment and diagnosis

All subjects underwent best-corrected visual acuity testing using the Early Treatment Diabetic Retinopathy Study (ETDRS) letter chart and full ophthalmic examination. Comprehensive retinal imaging included a volumetric dense raster OCT scan, SLO (fundus reflectance and autofluorescence), adaptive optics ophthalmoscopy and microperimetry. If inherited retinal disease was suspected, blood was obtained for genetic testing. Diagnosis was based on multimodal imaging and genetic test results if available.

### Mutation analysis and variant interpretation

Collection, extraction and storage of DNA was carried out as described in [25]. Proband DNA (50ng/μL) was analysed by Sanger sequencing of coding and flanking intronic regions of relevant genes: ABCC6 (OMIM 603234; NM_001171.5) for Case 1 and CYP4V2 (OMIM 608614; NM_207352.3) for Case 4. CYP4V2 was also analysed by MLPA for copy number variation. Family member DNA was subsequently analysed by targeted sequencing. Analysis of ABCC6 and CYP4V2 was performed by the Netherlands Institute for Neuroscience (NIN; Amsterdam) and Casey Eye Institute (CEI) Molecular Diagnostics Laboratory (Oregon, USA) respectively. Pathogenicity assessment of identified variants was performed by the Australian Inherited Retinal Disease Register and DNA Bank (AIRDR) in accordance with American College of Medical Genetics guidelines [26]: In silico predictions, allele frequencies, scientific literature and clinical diagnoses were considered accordingly. In silico predictions for missense variants were made using the online tools, Mutation Taster, Align GVGD [27], PolyPhen2 [28], and SIFT [29]. Allele frequency was assessed using ExAC Browser [30]. Pathogenicity assessment of Indel and nonsense variants was based on the likelihood of nonsense-mediated decay [31]. Variant information within online databases, Human Gene Mutation Database [32] and dbSNP [33] was also considered.

### OCT imaging

#### Instrument

We used a Heidelberg Spectralis spectral domain optical coherence tomography (SD-OCT) device (Heidelberg Engineering, Germany) to acquire sets of cross-sectional images of the human retina. The system operates at 40,000 A-scans per second and comprises a superluminescent diode, with a central wavelength of 870 nm and a bandwidth of 85 nm. It provides an axial resolution of 3.9 μm in tissue and the diameter of the beam spot at the retinal plane is around 14 μm (lateral resolution). The images were collected using the automatic retinal tracking (ART) mode to ensure that all B-scans within the imaging area of interest were aligned with NIR SLO image irrespective of eye movements. In this mode 9 B-scan frames were acquired and averaged at each retinal location. Motion artefacts and noise reduction were corrected in real time.

#### Scanning protocol

**Fig 1:**
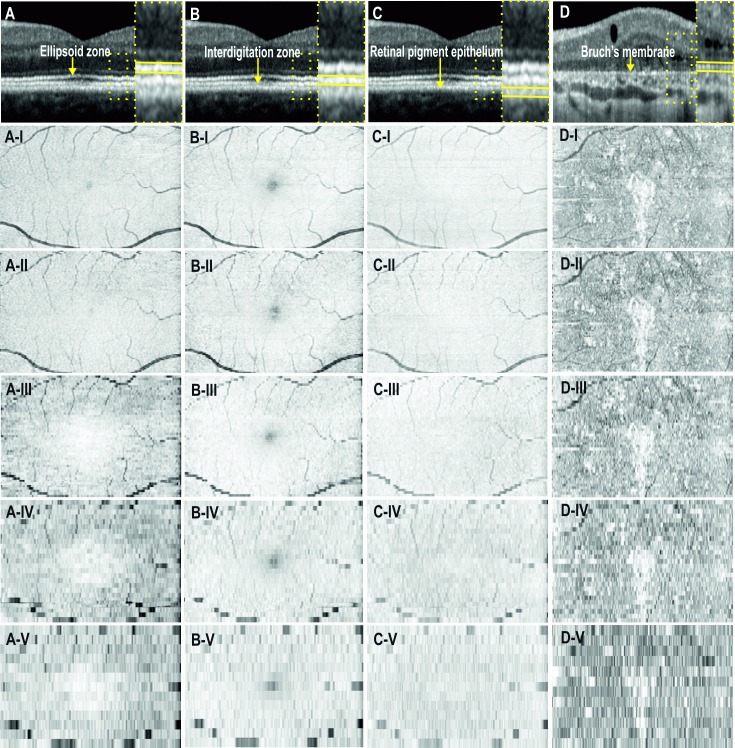
*En face* optical coherence tomography images of the ellipsoid zone (A-I:V), interdigitation zone (B-I:V), retinal pigment epithelium (C-I:V) and Bruch’s membrane (D-I:V). Images from the first 3 columns are from a normal subject and the last column is from a patient with Bietti crystalline dystrophy. The scanning protocol covers a 15° (horizontal) × 10° (vertical) field of view on the retina. Each row (I:V) corresponds to increasing separation between consecutive B-scans: 11, 30, 60, 120 and 240 μm.

The choice of the optimal clinically feasible scanning protocol is a compromise between the time required to obtain the volumetric dataset (to reduce subject’s fatigue), the field of view (FOV) and the spatial resolution of the volume scan. For patient comfort and reduced light exposure, the imaging time should be kept to a minimum by reducing the number of B-scans. However, sparsely-spaced B-scans will increase the chance of missing small retinal lesions. Conversely, densely-spaced B-scan will provide better resolution of the *en face* image but this increases acquisition time. To determine the optimal scanning protocol that balances acquisition time against adequate detail on *en face* OCT images, we created *en face* maps from OCT B-scans at varying separation. All available 3D protocols and the times required for OCT acquisition are listed in Table 2. Fig 1 shows the varying quality of reconstructed *en face* OCT images obtained from protocol 1 in which a 15° × 10° FOV in the central macular region is scanned using 5 different raster densities (ranging from 13 to 261 horizontal B-scan slices across the 2.9 mm vertical range).

**Table 2:** Volumetric scanning protocols available in Spectralis SD-OCT instrument.

	Horizontal scanning range		No. of A-scans / horizontal scan	Vertical scanning range		No. of averaged B-scans*	Distance between B-scans [μm]	Total imaging time**	
	[degree]	[mm]		[degree]	[mm]			Calculated [s]	Measured [s]
1.	**15**	4.4	768	**10**	2.9	261	11	45	
						**97****†**	**30**	**17**	**32–60**
						49	60	8	
						25	120	4	
						13	260	2	
2.	**20**	5.8	1024	**10**	2.9	261	11	60	
						97	30	22	
						49	60	11	
						25	120	6	
						13	260	3	
3.	**20**	5.8	1024	**15**	4.4	391	11	90	
						145	30	34	
						73	60	17	
						37	120	9	
						19	260	4	
4.	**20**	5.8	1024	**20**	5.8	521	11	120	
						193	30	45	
						97	60	23	
						**49†**	**120**	**11**	**39–60**
						25	260	6	
5.	**30**	8.7	1536	**15**	4.4	391	11	135	
						145	30	50	
						73	60	25	
						37	120	13	
						19	260	7	
6.	**30**	8.7	1536	**20**	5.8	521	11	180	
						193	30	67	
						97	60	34	
						49	120	17	
						**25****‡**	**260**	**9**	**18–30**
7.	**30**	8.7	1536	**25**	7.3	NP	NP	NA	
						**241†**	**30**	**83**	**200–240**
						122	60	42	
						**61‡**	**120**	**21**	**45–70**
						28	260	10	
8.	**30**	8.7	1536	**30**	8.7	NP	NP	NA	
						290	30	83	
						145	60	42	
						73	120	21	
						33	260	11	

*9 frames are averaged in each B-scan.

**The second last column presents total acquisition time calculated for each scanning protocol based on instrument scan rate, number of A-scans and B-scans and with assumption that there is no eye movement during the imaging. The last column presents our measured total imaging time for scanning protocols.

**†,‡ -** These scanning protocols are commonly used in clinical trials (**†**, black, bold) and routine care in our institution (‡, black, bold).

Fig 1 illustrates that a separation distance of 11–30 μm between B-scans provides the best *en face* OCT image quality in contrast to the pixelated image using a B-scan separation of 120–240 μm. Given that there is a 3-fold difference in acquisition time between 11 μm and 30 μm protocols we chose the 97 horizontal slice protocol for a small FOV of 15° × 10°, to limit the acquisition time to a maximum of 1 minute. For a larger FOV of 20° × 20°, a compromise in image quality may be needed by increasing separation between B-scans to 60 μm. This is because scanning at 30 μm separation results in an unacceptably long scanning time of approximately 2 minutes.

#### Segmentation of retinal layers and *en face* maps generation

**Fig 2:**
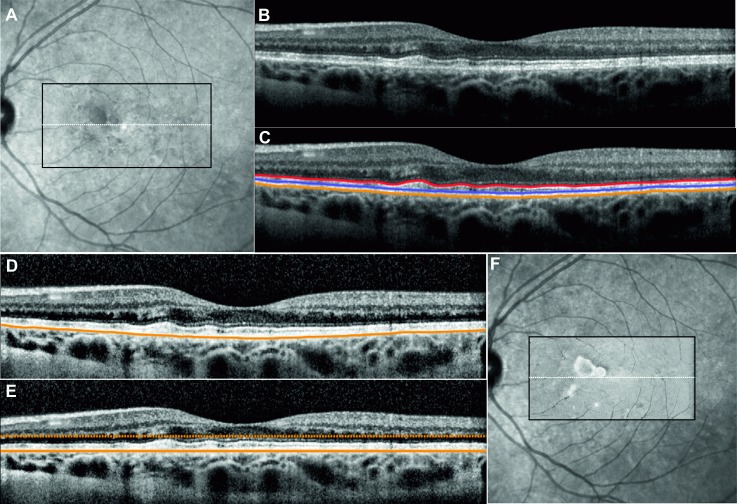
A 20° × 20° Near-infrared reflectance image NIR image (A) showing the region of the volumetric dense raster scan (black rectangle) and the location of the horizontal OCT B-scan (white line). Unprocessed OCT scan showed irregular elevation of the ellipsoid line due to a vitelliform lesion (B). Three layers of interest were segmented in this B-scan (C). The B-scan contrast is enhanced to reduce image quality variation between B-scans within the raster scan set (D). Using the base of the retinal pigment epithelium as a reference layer, the B-scan is flattened and a parallel boundary is chosen for creating a slab of pixels for generating the *en face* OCT image (E). The same procedure is repeated for consecutive B-scans to generate a reflectivity profile across the scanning area. The *en face* OCT image can be overlaid on the NIR image (F).

Custom-written software was used to analyze the OCT data from each subject. Automated segmentation methods based upon graph-search theory [12,34] were used to extract layers of interest in each image, after which an experienced masked observer verified the integrity of the automated segmentation of each of the boundaries of interest and manually corrected any segmentation errors as required. For this study, up to three retinal sublayers were segmented to extract a retinal region of interest including ellipsoid zone (EZ), interdigitation zone (IZ) and retinal pigment epithelium-Bruch’s membrane complex (RPE-BM). Fig 2 shows the image processing steps for an adult subject with acquired vitelliform lesions.

After retinal layer segmentation (Fig 2C), a contrast-limited adaptive histogram is applied to the image, to reduce inter B-scan variation in reflectivity (Fig 2D) [35]. The contrast can be limited to avoid amplification of noise. In the next step, the cross sectional image is flattened (Fig 2E) using the base of the RPE as a reference. To flatten the image, each A-scan (image column) is circularly shifted to set the reference boundary as the central part of the image. This flattening operation ensures the reference layer is located at the same depth across the volumetric scan. Thus, the *en face* OCT image can be created by extraction of the key region of interest within the volume matrix relative to the base of the RPE. Pixel layers above or below the reference layer can be cropped and averaged to generate one line of the *en face* OCT map. After repeating this for all of the B-scans in the volume scan set and ensuring correct spacing and alignment, a 2 dimensional reflectivity profile or *en face* OCT image is generated (Fig 2F). This profile can be derived from a single pixel (a plane) or stacks of pixels (a slab) across the macular region. For a slab of pixels, a range of operation (*i*.*e*. mean, sum, median, minimum or maximum) can be used to generate the reflectivity intensities of the vertical stack of pixels at each location of the *en face* OCT image. The choice of these visualization techniques depends on the thickness of the slab and the pathological feature. For example, minimum intensity projection may be used to highlight pathology that reduces reflectivity within a slab that is uniformly hyper-reflective (EZ, IZ or RPE) whereas maximum intensity project may be used to highlight hyper-reflective lesions within the hypo-reflective inner and outer nuclear layers. Mean intensity projection is the most common method to generate *en face* maps and is used in this study. The EZ *en face* map is generated from pixel layers between the automatically segmented top boundary of EZ and manually selected bottom boundary of EZ (~5 pixels below EZ contour). IZ *en face* map is generated from pixel layers between the automatically segmented bottom boundary of IZ and manually selected top boundary of IZ (~5 pixels above IZ contour). RPE *en face* map is generated from pixel layers between the automatically segmented bottom boundary of RPE and manually selected top boundary of RPE (~8 pixels above RPE contour).

#### Heidelberg Eye Explorer software for *en face* map generation

The Heidelberg Eye Explorer software (Heidelberg Eye Explorer, version 1.9.10.0; Heidelberg Engineering) allows for *en face* visualization of outer retinal layers. However, segmentation lines for generating *en face* OCT image can only be aligned to automated segmentation line demarcating BM or internal limiting membrane. Manual adjustment in these segmentation lines is possible, however very much time consuming.

### SLO imaging

Confocal scanning laser ophthalmoscopy was used to obtain 30° fields of view retinal (FOV) images (Heidelberg Engineering, Germany). We used 3 spectra: near-infrared reflectance (at 820 nm), blue-light autofluorescence (excitation at 488 nm and emission >510 nm), and infrared autofluorescence (excitation at 785 nm and emission: 805–840 nm).

### Microperimetry

Microperimetry was performed prior to any retinal imaging or examination using the fundus-controlled microperimeter MAIA (Centervue, Padova, Italy). Pupils were dilated with tropicamide 1% and phenylephrine 2.5%. Testing was conducted in a darkened room after 5 minutes of dark adaptation. A 4–2 staircase strategy was used to determine retinal sensitivity thresholds to the nearest 1dB.

### Adaptive optics imaging

Perifoveal cone photoreceptor tips were visualized using an adaptive optics flood-illumination ophthalmoscope (AO-FIO, rtx1, Imagine Eyes, Orsay, France). During a single measurement, 40 images are acquired in the same location over 4 seconds. Increased signal-to-noise ratio in the final AO image is achieved by selection, registration and averaging of up to 40 raw AO images. Each AO image covers a 4° × 4° FOV (750 × 750 pixels–oversampled to 1500 × 1500 pixels) region in the retina; approximately 1.2 × 1.2 mm. To cover a larger area of the retina 8 to 20 adjacent AO image frames were acquired with a 1°–2° region of overlap. Foveal cone tips are not visible on the rtx1 device because the resolution of the system is only 250 line pairs per mm. Therefore, cone identification within 2.5° from the foveal center is unreliable and not suitable for quantification [36].

Photoreceptors within each AO-FIO images were identified using the AO Detect software provided by the instrument manufacturer. This software automatically detects the central coordinates of small circular spots whose brightness are higher than the surrounding background level. Results are provided in the form of two images, one that visualizes the cones and the second that presents a color-coded density map of cones. These image sets were then montaged using the MosaicJ plugin available in ImageJ software [37].

### Comparison and registration between all modalities

Different imaging modalities were compared by alignment against retinal vascular patterns. We compared: 1) NIR and AF with *en face* OCT images for each outer retinal layer; 2) AO-FIO images with *en face* OCT images of the EZ and IZ; and 3) microperimetry with adaptive optics and *en face* OCT images.

## Results

### Healthy control subjects

**Fig 3:**
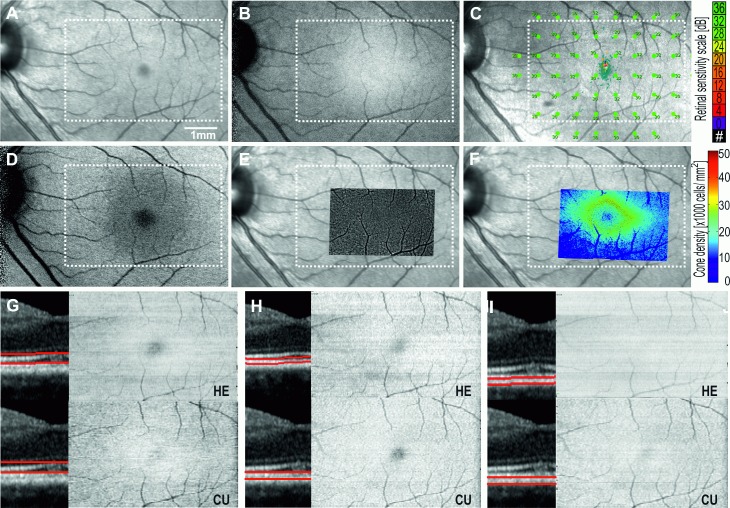
Multimodal imaging of the left eye of a healthy subject showing normal 15° × 10° Near-infrared reflectance NIR (A), infrared autofluorescence IRAF (B), microperimetry (C), blue-light autofluorescence BAF (D), adaptive optics flood illumination ophthalmoscopy AO-FIO cone montage and density maps overlaid on NIR (E,F), and *en face* OCT maps of the ellipsoid zone EZ (G), interdigitation zone IZ (H), and retinal pigment epithelium RPE (I). HE, Heidelberg Eye Explorer software (Heidelberg Engineering); CU, Custom-built software using graph-search theory algorithm. White dotted rectangle corresponds to region for which *en face* maps are generated.

Fig 3 shows images obtained from the left eye of a 33 year old female control subject. All images displayed homogeneous signal without significant variation. Retinal vasculature visibility was enhanced in the EZ and IZ *en face* OCT images due to shadowing effects. The foveal center appeared hypo-reflective, relative to the surrounding perifoveal retina in NIR, EZ and IZ *en face* OCT images due to elevation and attenuation of the EZ and IZ lines at the fovea externa.

### Case 1: Well-defined lesion on SLO but not on OCT B-scan

**Fig 4:**
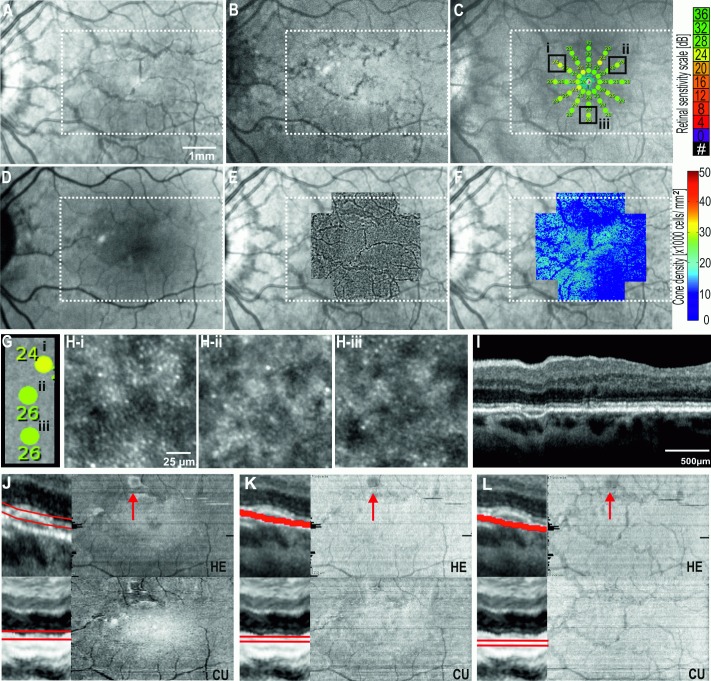
Multimodal imaging showing the left eye of Case 1: angioid streaks secondary to pseudoxanthoma elasticum. Near-infrared reflectance NIR (A), infrared autofluorescence IRAF (B), microperimetry (C), blue-light autofluorescence BAF (D), and adaptive optics flood illumination ophthalmoscopy AO-FIO cone montage and density maps overlaid on NIR image (E, F) of the central 15° × 10° showing the angioid streaks as irregular linear hypo-reflective lesions. The occasional streaks showing hypo-autofluorescence, relative scotoma in regions unaffected by angioid streaks and cone tip reflexes within the region of angioid streaks. Microperimetry overlaid on cone tip reflex montage (G) showed reduced cone densities compared with normative values, both in regions with reduced (H:I) and normal sensitivity (H:II-III). B-scans flattened to the base of retinal pigment epithelium RPE (I) are used for generating e*n face* OCT images of the ellipsoid zone (J), apical portion of the RPE (K) and basal portion of the RPE (L). Inaccuracy in automated segmentation of the basal RPE by HE resulted in artefact in the ellipsoid zone and RPE *en face* OCT images (red arrow in K, L and M). The linear pattern of angioid streaks were only visible in the *en face* OCT image derived from the basal portion of the RPE (L). HE, Heidelberg Eye Explorer software (Heidelberg Engineering); CU, Custom-built software using graph-search theory algorithm. White dotted rectangle corresponds to region for which *en face* OCT images are generated.

A 42 year old female was referred for assessment of asymptomatic retinal lesions. Best-corrected visual acuity was 87 and 86 letters in the right and left eyes respectively. Dilated fundus examination revealed irregular, linear, dark red streaks extending away from the optic disc and peau d’orange lesions temporally in each eye (Fig 4). Given the history of presumed pseudoxanthoma elasticum in her sister, genetic testing was performed.

Two pathogenic changes in the ABCC6 gene were identified, which together would result in the complete absence of the ABCC6 protein due to nonsense mediated decay. The previously reported nonsense variant, c.3421C>T (rsID: rs72653706; HGMD: CM001043) is considered a rare pathogenic allele encoding a premature stop codon (p.Arg1141*), whilst the large, novel deletion, c.2996_4208del would result in a frameshift and premature truncation of the encoded protein product (p.Ile1000Trpfs*60).

NIR imaging enhanced visualization of angioid streaks as hypo-reflective (dark) branching lines radiating from the optic disc and hypo-reflective spots in the temporal and superior perifoveal region. Some of the streaks in the fovea were also visible on IRAF but most streaks were not seen on BAF imaging except in regions of RPE loss. AO-FIO demonstrated relatively densely packed cone tip reflexes even within the region of angioid streaks. However, microperimetry showed relative scotoma (< 27 dB) within the fovea even in regions unaffected by angioid streaks as seen on AO-FIO and normal appearance and thickness of the outer retinal layers on OCT scan. *En face* OCT images of the EZ and RPE were generated using both the built-in 3D-view module in the commercial software and custom segmentation software.

Commercial software was able to align segmentation lines with the base of the RPE except in the region of a pigment epithelial detachment (PED). This precluded accurate visualization of EZ and RPE *en face* OCT images overlying the PED. In contrast, our custom segmentation algorithm flattened the B-scan to the base of the RPE, thus negating the effect of the RPE elevation at the PED lesion. Compared to commercial software, our custom software produced an *en face* OCT image of the basal portion of the RPE layer that more closely resembles the pattern of angioid streaks seen with NIR and AO-FIO (Fig 4).

### Case 2: Subtle lesions on both SLO and OCT B-scan

**Fig 5:**
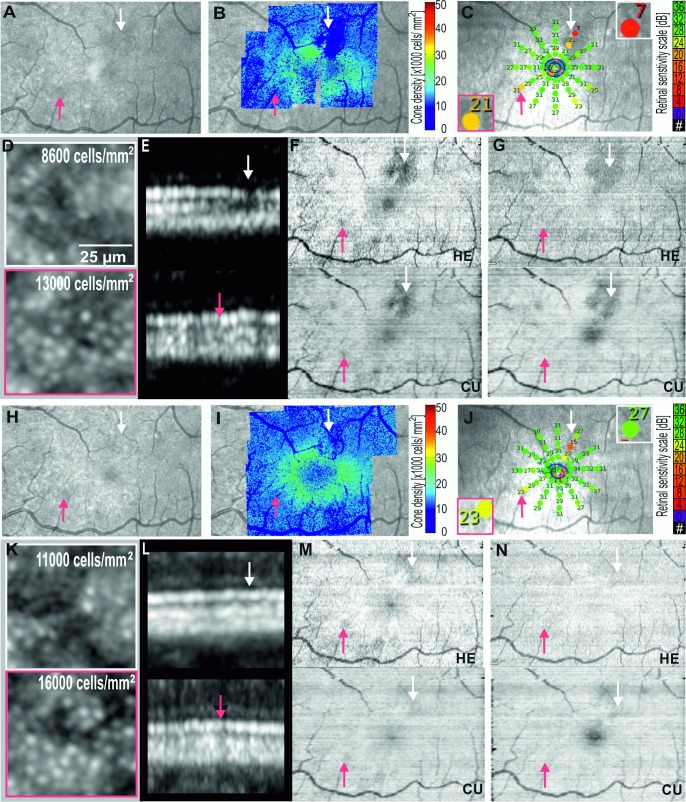
Multimodal imaging showing the left eye of Case 2: acute macular neuroretinopathy at presentation (A-G), and 6 months (H-N) of follow-up. Near-infrared reflectance NIR (A,H), adaptive optics flood illumination ophthalmoscopy AO-FIO density map overlaid on NIR (B,I,) and magnified cone images (D,K), microperimetry (C,J), OCT B-scans in the region of relative scotoma (E,L) and *en face* OCT images of ellipsoid (F,M), and interdigitation zones (G,N) showed partial recovery. Both commercial and custom software were able to demonstrate the topography of ellipsoid and interdigitation zone injury.

A 49 year old female presented with a 4-week history of acute onset paracentral scotoma in her left eye. Best-corrected visual acuities were 85 and 71 letters in the right and left eyes respectively. Anterior segment examination was normal. Fundus examination showed a slightly reddish wedge-shaped lesion superior to the fovea in the left eye and this corresponded to a hypo-reflective lesion on NIR imaging. AF was normal. OCT B-scan revealed focal loss of IZ signal and attenuation of the EZ integrity in the region of the wedge-shape lesion (Fig 5). There were no other obvious retinal or choroidal changes adjacent to these regions. Microperimetry demonstrated two loci with reduced retinal sensitivity in the supero-temporal fovea corresponding to the wedge-shaped lesion seen on NIR imaging. However, there were also other test loci infero-nasally and infero-temporally at 3° eccentricity with reduced retinal sensitivity. AO-FIO demonstrated loss of cone reflexes not only within the wedge-shaped lesion but also at other regions that coincided with relative scotoma (Fig 5). *En face* OCT images of EZ and IZ showed low reflectivity profile that corresponded to the regions of cone reflex loss on AO-FIO. Hence, there were several lesions that were visible on the *en face* OCT image which were not on NIR.

On follow up at 6 months, serial OCT demonstrated partial recovery of the integrity of the EZ and IZ in the affected regions and improved visualization of cone tip reflexes on AO-FIO. There was improvement in retinal sensitivity from 7 to 27 dB and some of the defects seen on *en face* OCT images of EZ and IZ resolved. The main wedge-shaped lesion also reduced in size over the 6 months (Fig 5). There was no significant difference in the *en face* OCT images generated by commercial and our custom software in illustrating the course of AMN recovery since retinal segmentation were almost identical.

### Case 3: No obvious lesions in either SLO or OCT

**Fig 6:**
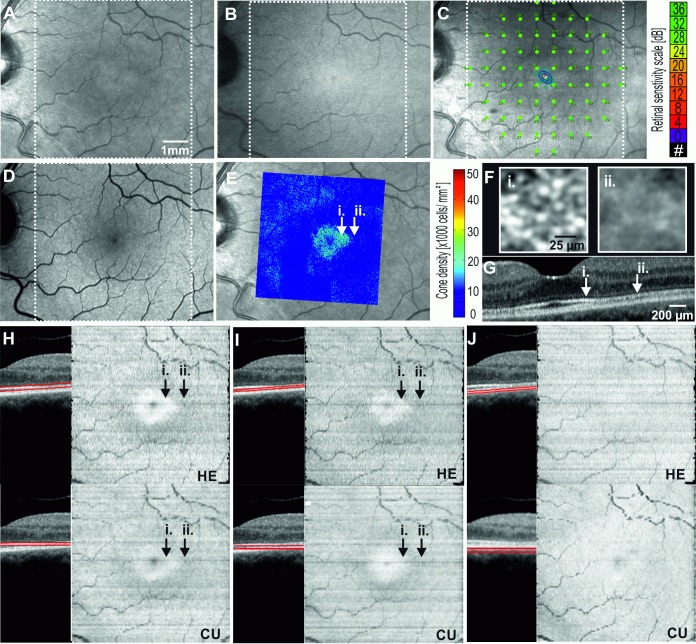
Multimodal imaging showing the left eye of Case 3: early hydroxychloroquine toxicity. 20° × 20° Near-infrared reflectance NIR (A) reveals a subtle hyper-reflective foveal signal while infrared autofluorescence IRAF (B), microperimetry (C) and blue-light autofluorescence BAF (D) are within normal limits. Adaptive optics flood illumination ophthalmoscopy AO-FIO density map overlaid on NIR (E) and cone image (F) reveals a transition zone in the parafovea where cone reflexes are lost (E). OCT B-scan (G) through the foveal center demonstrates preserved (i.) and attenuated (ii.) interdigitation zone at parafovea. The *en face* OCT image reveals a para- and perifoveal concentric zone of reduced reflectivity from the ellipsoid zone EZ (H) and the interdigitation zone IZ (I). Retinal pigment epithelium RPE layer *en face* OCT appeared normal (J). In this case, HE is able to accurately identify the contour of Bruch’s membrane and hence the *en face* OCT images are similar to the one generated by our custom software (CU). White dotted square corresponds to region for which *en face* maps are generated.

A 53 year old female with rheumatoid arthritis presented for screening of hydroxychloroquine (HCQ) toxicity. She had been using HCQ for 16 years with a cumulative dose of 2275 mg and an daily dose of 6.45 mg/kg body weight. A 10–2 Humphrey visual field (HVF) test with a white target demonstrated a partial ring scotoma superior to fixation in both eyes. There was no abnormality on AF. NIR imaging showed a subtle ring of relative hyper-reflectance in the foveal zone (Fig 6).

AO-FIO revealed absence of normal cone reflexes in the parafoveal region compared to an adjacent area of well-visualized cone reflexes closer to the fovea. Structural abnormality was confirmed on OCT, with subtle attenuation of the IZ and EZ. *En face* OCT images of the EZ and IZ layers revealed that this attenuation spared the foveal zone at both levels. The reflectivity profile of the RPE was normal throughout the corresponding retinal region. The patient was advised to cease HCQ.

### Case 4: Well-defined lesion on OCT but not on SLO

A 51 year old female presented with a 15 year history of nyctalopia and peripheral vision loss. Her parents and her brothers were unaffected. The best-corrected visual acuities were 71 and 75 letters in the right and left eyes respectively. There were limbal crystalline deposits in both corneas and numerous refractile subretinal deposits. Widespread retinal and choroidal atrophy were noted. Genetic testing was performed to confirm Bietti crystalline dystrophy (BCD).

Two previously reported, biallelic missense variants were found in the CYP4V2 gene. The respective paternally and maternally inherited variants, c.1168C>T (rsID: rs776616377; HGMD: CM119423) and c.1198C>T (rsID: rs138444697; HGMD: CM074768), result in arginine to cysteine amino acid substitutions, p.Arg390Cys and p.Arg400Cys, respectively. These rare variants are predicted to be disease-causing by in silico modelling and taken together, are considered the likely primary disease variants.

**Fig 7:**
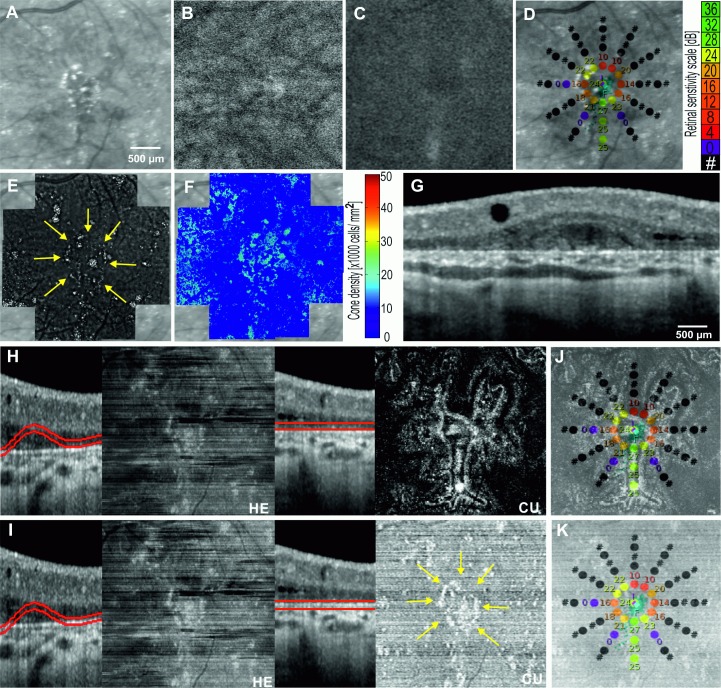
Multimodal imaging showing the left eye of Case 4: Bietti crystalline dystrophy. Although crystals are seen, the boundary of retinal degeneration is not clearly visualized on Near-infrared reflectance NIR (A), infrared autofluorescence IRAF (B) or blue-light autofluorescence BAF (C). Microperimetry (D) demonstrates preserved retinal sensitivity within 1° of the center of fixation indicating the presence of foveal photoreceptor cells supported by an island of RPE. Crystals are also visualized with adaptive optics flood-illumination ophthalmoscopy AO-FIO (E) but the poor signal from cones precluded AO-FIO cone density mapping (F). The OCT B-scans (G) reveal atrophy of the RPE and outer retinal layers. As a direct consequence, the errors in automated segmentation of the Bruch’s membrane by HE lead to artefacts in the *en face* OCT images of the ellipsoid-RPE slab (H-HE) and the Bruch’s membrane (I-HE). Our custom algorithm was able to generate ellipsoid-RPE (H-CU) and Bruch’s membrane (I-CU) *en face* OCT images. The well-defined central island of ellipsoid-RPE correlated well with the region of retinal sensitivity on microperimetry (J). The distribution of crystals seen on AO imaging (E, yellow arrows) mirrors the lesions seen on *en face* OCT of Bruch’s membrane (I-CU, K, yellow arrows).

NIR imaging demonstrated small punctate hyper-reflective lesions corresponding to the retinal crystals (Fig 7). However, neither NIR nor AF were able to reveal the boundary of the preserved foveal island of RPE. AO-FIO was unable to illustrate cone tip reflex due to poor image signal related to interference from crystalline deposits at the level of BM. Preservation of cone photoreceptors was inferred from the presence of retinal sensitivity on microperimetry at the foveal center. This island of vision was surrounded by a ring of dense scotoma encroaching into test loci at 1° from fixation in the right eye, and 2° from fixation in the left eye. There was extensive outer retinal and RPE atrophy on OCT beyond 2° of eccentricity. Electrophysiology demonstrated residual photoreceptor function, more so in the left eye (not shown). *En face* OCT images of the EZ and RPE were generated using both the built-in 3D-view module in the Heidelberg software and our custom segmentation software.

Owing to the disrupted outer retinal architecture, the commercial software was not able to automatically identify the contour of BM accurately, resulting in *en face* OCT images that display jumbled information from different retinal layers. To visualize EZ-RPE, OCT B-scans were flattened with reference to the BM so that a parallel slab of the EZ and RPE layers can be isolated for creating *en face* OCT image (Fig 7). A central island of preserved RPE reflectivity was demonstrated using custom software and the boundary of this region encompassed retinal loci with preserved sensitivity. In this case, AO-FIO was unable to verify the preservation of photoreceptors because foveal cones are not resolved by the rtx1 camera system.

## Discussion

We developed a graph-search theory algorithm-based method to generate *en face* OCT images of the outer retinal layers. In a pilot study of normal eyes, we demonstrated the rationale for selecting an image acquisition protocol for deriving optimal *en face* OCT images. In four unique cases of retinal diseases, we illustrated the utility of *en face* OCT images generated by our custom software in clarifying apparent mismatch between NIR, FAF, OCT, AO-FIO and microperimetry.

We demonstrated that *en face* OCT image resolution is improved by reducing the separation between B-scans. In recognition of this relationship, the tested commercial software will only generate *en face* OCT images from volume scans with OCT B-scan separation of 60 μm or less. For a smaller scanning area, a B-scan separation of 30 μm is feasible but for a larger area, a B-scan separation of 60 μm is more appropriate due to prolonged image acquisition time. In support of our recommendation, Hariri *et al*. also demonstrated good inter-grader agreement in lesion size measurement on the EZ *en face* OCT image can be achieved by 20° × 20° volume scans with 60 μm B-scan separation [38]. However, it is not known if the repeatability of *en face* OCT image parameters is dependent on scanning density. We noted that some *en face* OCT images had horizontal bands of reduced reflectivity and this is partially corrected by our custom software through the incorporation of an additional step of contrast enhancement of the B-scan set. However, standardized techniques to minimize the variation in B-scan reflectivity across the scanning area will be required to enable inter-session and inter-individual comparison of quantitative *en face* OCT image analysis.

Previous reports have demonstrated that angioid streaks co-localized with focal discontinuity in the BM with varying effect on the overlying RPE layer [39]. However, not all angioid streaks seen on NIR correspond to a visible abnormality in the RPE-BM complex on SD-OCT [40]. We were able to demonstrate co-localization of angioid streaks on NIR with hypo-reflective pixels within the basal portion of apparently normal RPE-BM complex [41]. This is consistent with the understanding that both RPE and BM contributes to the “RPE” hyper-reflective band on SD-OCT. Moreover, angioid streaks were also visible on IRAF and this is consistent with previous histological studies that showed reduced melanin in the RPE cells overlying these streaks. Given that melanin within the RPE is predominantly located in the apical side, it is surprising that *en face* OCT of the apical pixels of the RPE-BM complex did not demonstrate angioid streaks. RPE over these streaks may degenerate over time resulting in loss of RPE and hypo-autofluorescence on BAF. We also demonstrated subtle reduction in foveal retinal sensitivity in regions unaffected by angioid streaks. Although cone tip reflexes were seen within the angioid streaks, there was an overall reduction in cone density. Therefore, we postulate that the reduced retinal function in the foveal region may be related to ectopic mineralization of the BM rather than the actual presence of angioid streaks.

Both AMN and early HCQ toxicity affect the IZ with variable EZ involvement. Outer nuclear layer thickness may be reduced with more severe damage to the photoreceptors. Although clinically visible as a dark red wedge-shaped lesion pointing towards the fovea, we demonstrated that subclinical lesions in AMN can surround other portion of the parafovea. Prompted by the unexpected finding of retinal sensitivity loss in test loci remote from the wedge-shaped lesion, we identified and confirmed satellite lesions through AO-FIO and *en face* OCT images of the IZ and EZ. These satellite lesions were not visible on OCT B-scan because there was only minimal attenuation of IZ reflectivity. Follow up *en face* OCT image, AO-FIO and microperimetry demonstrate partial recovery of function and structure over a 6 month period. In contrast to the case reported by Affortit *et al*., we illustrated the utility of *en face* OCT imaging in identifying AMN lesions that were not visible on NIR [42]. In the case of suspected HCQ toxicity based on perifoveal loss of sensitivity on Humphrey visual field test, SD-OCT showed relatively intact EZ but subtle thinning of IZ in the parafoveal region. There was no classical flying saucer sign of HCQ toxicity in which loss of EZ is accompanied by gross thinning of the outer nuclear layer. Although BAF showed no abnormality, NIR demonstrated a zone of relative hyper-reflectivity within the fovea. The peri- and parafoveal localization of IZ pathology was confirmed by the AO-FIO finding of sharp reduction in cone density. The loss of reflectivity of EZ and IZ in the perifoveal region as seen on the *en face* OCT image correlated with the perifoveal hypo-reflectivity on NIR. Unlike the case presented by Itoh *et al*., we demonstrated abnormalities in both IZ and EZ *en face* OCT images generated from relatively normal appearing SD-OCT B-scans [43].

In many rod cone dystrophies, the region of residual foveal photoreceptor can be readily quantified by IRAF or BAF signal arising from the residual island of RPE. However, in the case of BCD, the residual island was not readily visible on either form of AF imaging or NIR imaging despite clear evidence of preservation on SD-OCT B-scan. Unfortunately, AO-FIO systems also do not have adequate resolution to visualize the cones within 2° of the foveal center. To quantify the area of residual island of RPE or photoreceptors, we created EZ-RPE *en face* OCT images to highlight the boundaries of this region. The pattern of preserved reflectivity of these layers co-localized with the distribution of preserved retinal sensitivities on microperimetry. This method of visualizing residual photoreceptors in BCD could be a useful tool in corroborating the functional deficits seen on microperimetry and monitoring disease progression rates. Visualization of the crystalline deposits in BCD on OCT B-scans can also be enhanced by *en face* OCT image of the BM. However, these crystals only occupy a small number of pixels and therefore AO-FIO may be more suitable given the superior resolution. Nevertheless, our *en face* OCT image of the RPE-BM complex confirmed that the location of these crystalline deposit is at the BM as previously reported [44]. It may be useful to monitor the topographic changes in the distribution of crystals occurring at BM with serial *en face* OCT imaging as it has been described previously that retinal atrophy is preceded by disappearance of the crystals [45].

There are several limitations in our method of generating *en face* OCT images. Firstly, high density volumetric OCT scanning is necessary (maximum of 60 micron separation to generate reasonable quality *en face* OCT image). For some patients the imaging time required to collect volumetric datasets with inter-B-scab distances equal to or less than 60 microns can be unacceptably long and this can result in more motion artifacts. Second, despite improved segmentation accuracy using the graph-search theory algorithm, errors in identifying BM or the base of RPE can still occur. Amongst subjects presented in this paper, manual correction of segmentation errors was necessary in Cases 1(angioid streaks) and 4 (Bietti crystalline dystrophy). For Case 1, 8 B-scans from the set of 97 had to be corrected at the level of ellipsoid zone, no correction was required for the IZ or RPE. Using Heidelberg software, 35 B-scans required manual correction for accurate generation of EZ and IZ maps and 2 B-scans at level of RPE. For case 4, 22 B-scans from the set of 271 had to be corrected at the level of Bruch Membrane using our software and all B-scans had to be corrected using commercial software.

The graph-search theory algorithm may be adapted to take into account the effect of the pathology on the OCT image [46,47], but despite the application of contrast-limited adaptive histogram to the B-scans to reduce variations in image quality, artefacts in the *en face* OCT image cannot be entirely removed. This leads to difficulty in standardization of the image brightness and defining pixel layers above BM that should be used to generated RPE, IZ and EZ *en face* OCT image. Finally, we only have a small number of patients but the purpose of our study is to illustrate the potential clinical utility rather than to determine the prevalence of specific lesions. Further studies are required to examine the feasibility of incorporating this method of visualizing OCT scans into routine clinical care given the prolonged time required to process the B-scans and generate the *en face* OCT images.

## Conclusions

We have developed an image processing tool that provides more flexibility during the reconstruction of *en face* OCT images. We illustrate the clinical utility of the software in patients with subtle lesions on OCT and SLO but clear abnormality on AO-FIO and microperimetry. Future work is needed in standardizing image reflectivity such as introducing an attenuation coefficient of the tissue [48] which will assist with quantification of reflectivity of each layer and longitudinal analysis of age related and pathological changes over time.

## References

[1] Novais EA, Baumal CR, Sarraf D, Freund KB, Duker JS. Multimodal imaging in retinal disease: A Consensus Definition. Ophthalmic Surg Lasers Imaging Retina. 2016;47(3):201–5. 10.3928/23258160-20160229-01 26985792

[2] Girkin C. Principles of confocal scanning laser ophthalmoscopy for the clinician. The Essential HRT Primer. 2005;1–9.

[3] Wojtkowski M. High-speed optical coherence tomography: basics and applications. Appl Opt. 2010;49(16): 30–61. 10.1364/AO.49.000D30 20517358

[4] Francis AW, Wanek J, Lim JI, Shahidi M. Enface thickness mapping and reflectance imaging of retinal layers in diabetic retinopathy. PLoS One. 2015;10(12):1–15. 10.1371/journal.pone.0145628 PMC4699197 26699878

[5] Yang Q, Reisman CA Wang Z, Fukuma Y, Hangai M, Yoshimura N, et al Automated layer segmentation of macular OCT images using dual-scale gradient information. Opt Express. 2010;18(20):21293–307. 10.1364/OE.18.021293 20941025 PMC3101081

[6] Cabrera Fernández D, Salinas HM, Puliafito CA. Automated detection of retinal layer structures on optical coherence tomography images. Opt Express. 2005;13(25):10200–16. 19503235 10.1364/opex.13.010200

[7] Ishikawa H, Stein DM, Wollstein G, Beaton S, Fujimoto JG, Schuman JS. Macular segmentation with optical coherence tomography. Invest Ophthalmol Vis Sci. 2005;46(6):2012–7. 10.1167/iovs.04-0335 15914617 PMC1939723

[8] Fabritius T, Makita S, Miura M, Myllylä R, Yasuno Y. Automated segmentation of the macula by optical coherence tomography. Opt Express. 2009;17(18):15659–69. 10.1364/OE.17.015659 19724565

[9] Mishra A, Wong A, Bizheva K, Clausi DA. Intra-retinal layer segmentation in optical coherence tomography images. Opt Express. 2009;17(26):23719–28. 10.1364/OE.17.023719 20052083

[10] Wojtkowski M, Sikorski BL, Gorczynska I, Gora M, Szkulmowski M, Bukowska D, et al Comparison of reflectivity maps and outer retinal topography in retinal disease by 3-D Fourier domain optical coherence tomography. Opt Express. 2009;17(5):4189–207. 19259255 10.1364/oe.17.004189 PMC2743201

[11] Alonso-Caneiro D, Read SA, Collins MJ. Automatic segmentation of choroidal thickness in optical coherence tomography. Biomed Opt Express. 2013;4(12):2795–812. 10.1364/BOE.4.002795 24409381 PMC3862153

[12] Chiu SJ, Li XT, Nicholas P, Toth CA, Izatt JA, Farsiu S. Automatic segmentation of seven retinal layers in SDOCT images congruent with expert manual segmentation. Opt Express. 2010;18(18):19413–28. 10.1364/OE.18.019413 20940837 PMC3408910

[13] Mohammad F, Wanek J, Zelkha R, Lim JI, Chen J, Shahidi M. A method for en face OCT imaging of subretinal fluid in age-related macular degeneration. J Ophthalmol. 2014;2014:1–6. 10.1155/2014/720243 PMC4244942 25478209

[14] Gorczynska I, Srinivasan VJ, Vuong LN, Chen RWS, Liu JJ, Reichel E, et al Projection OCT fundus imaging for visualising outer retinal pathology in non-exudative age-related macular degeneration. Br J Ophthalmol. 2009;93(5):603–9. 10.1136/bjo.2007.136101 18662918 PMC2743133

[15] Haeker M, Sonka M, Kardon R, Shah VA, Wu X, Abràmoff MD. Automated segmentation of intraretinal layers from macular optical coherence tomography images. Proc SPIE 6512, Med Imaging 2007 Image Process. 2007;6512:651214.

[16] Garvin MK, Abràmoff MD, Wu X, Member S, Russell SR, Burns TL, et al Automated 3-D intraretinal layer segmentation of macular spectral-domain optical coherence tomography images. IEEE Trans Med Imaging. 2009;28(9):1436–47. 10.1109/TMI.2009.2016958 19278927 PMC2911837

[17] Yazdanpanah A, Hamarneh G, Smith BR, Sarunic M V. Segmentation of intra-retinal layers from optical coherence tomography images using an active contour approach. IEEE Trans Med Imaging. 2011;30(2):484–96. 10.1109/TMI.2010.2087390 20952331

[18] Vermeer KA, van der Schoot J, Lemij HG, de Boer JF. Automated segmentation by pixel classification of retinal layers in ophthalmic OCT images. Biomed Opt Express. 2011;2(6):1743–56. 10.1364/BOE.2.001743 21698034 PMC3114239

[19] Sallo FB, Peto T, Egan C, Wolf-Schnurrbusch UEK, Clemons TE, Gillies MC, et al En face OCT imaging of the IS/OS junction line in type 2 idiopathic macular telangiectasia. Invest Ophthalmol Vis Sci. 2012;53(10):6145–52. 10.1167/iovs.12-10580 22899757 PMC4608676

[20] Puche N, Querques G, Blanco-Garavito R, Zerbib J, Gherdaoui F, Tilleul J, et al En face enhanced depth imaging optical coherence tomography features in adult onset foveomacular vitelliform dystrophy. Graefe’s Arch Clin Exp Ophthalmol. 2014;252(4):555–62. 24158372 10.1007/s00417-013-2493-2

[21] Heiferman MJ, Fernandes JK, Munk M, Mirza RG, Jampol LM, Fawzi AA. Reticular pseudodrusen on infrared imaging are topographically distinct from subretinal drusenoid deposits on en face optical coherence tomography. Retina. 2015;35(12):2593–603. 10.1097/IAE.0000000000000666 26131588 PMC4658323

[22] Chen Q, Leng T, Zheng LL, Kutzscher L, De Sisternes L, Rubin DL. An improved optical coherence tomography-derived fundus projection image for drusen visualization. Retina. 2014;34(5):996–1005. 10.1097/IAE.0000000000000018 24177190

[23] Pilotto E, Guidolin F, Convento E, Antonini R, Stefanon FG, Parrozzani R, et al En face optical coherence tomography to detect and measure geographic atrophy. Invest Ophthalmol Vis Sci. 2015;56(13):8120–4. 10.1167/iovs.15-17366 26720464

[24] Mesiwala NK, Shemonski N, Sandrian MG, Shelton R, Ishikawa H, Tawbi H a, et al Retinal imaging with en face and cross-sectional optical coherence tomography delineates outer retinal changes in cancer-associated retinopathy secondary to Merkel cell carcinoma. J Ophthalmic Inflamm Infect. 2015;5(1):1–7. 26285790 10.1186/s12348-015-0053-0 PMC4540718

[25] De Roach JN, Mclaren TL, Paterson RL, O’Brien EC, Hoffmann L, Mackey DA, et al Establishment and evolution of the Australian Inherited Retinal Disease Register and DNA Bank. Clin Exp Ophthalmol. 2013;41(5):476–83. 10.1111/ceo.12020 23078154

[26] Richards S, Aziz N, Bale S, Bick D, Das S, Gastier-Foster J, et al Standards and guidelines for the interpretation of sequence variants: a joint consensus recommendation of the American College of Medical Genetics and Genomics and the Association for Molecular Pathology. Genet Med. 2015;17(5):405–23. 10.1038/gim.2015.30 25741868 PMC4544753

[27] Tatvtigian S, Byrnes G, Godgar D, Thomas A. Classification of Rare Missense Substitutions, Using Risk Surfaces, With Genetic- and Molecular-Epidemiology Applications. Hum Mutat. 2008;29(11):1342–54. 10.1002/humu.20896 18951461 PMC3938023

[28] Adzhubei I, Schmidt S, Peshkin L, Ramensky V, Gerasimova A, Bork P, et al A method and server for predicting damaging missense mutations. Nat Methods. 2010;7(4):248–9. 10.1038/nmeth0410-248 20354512 PMC2855889

[29] Ng PC, Henikoff S. Predicting Deleterious Amino Acid Substitutions. Genome Res. 2001;11(5):863–74. 10.1101/gr.176601 11337480 PMC311071

[30] The Exome Aggregation Consortium [Internet]. Available from: http://exac.broadinstitute.org/.

[31] Maquat LE. Nonsense-Mediated mRNA Decay: Splicing, Translation And mRNP Dynamics. Nat Rev Mol Cell Biol. 2004;5(2):89–99. 10.1038/nrm1310 15040442

[32] Stenson PD, Mort M, Ball E V., Shaw K, Phillips AD, Cooper DN. The Human Gene Mutation Database: Building a comprehensive mutation repository for clinical and molecular genetics, diagnostic testing and personalized genomic medicine. Hum Genet. 2014;133(1):1–9. 10.1007/s00439-013-1358-4 24077912 PMC3898141

[33] Sherry ST, Ward MH, Kholodov M, Baker J, Phan L, Smigielski EM, et al dbSNP: the NCBI database of genetic variation. Nucleic Acids Res. 2001;29(1):308–11. 11125122 10.1093/nar/29.1.308 PMC29783

[34] Read SA, Collins MJ, Vincent SJ, Alonso-Caneiro D. Macular retinal layer thickness in childhood. Retina. 2015;35(6):1223–33. 10.1097/IAE.0000000000000464 25650708

[35] Pisano ED, Zong S, Hemminger BM, DeLuca M, Johnston RE, Muller K, et al. Contrast limited adaptive histogram equalization image processing to improve the detection of simulated spiculations in dense mammograms. J Digit Imaging. 1998;11(4):193–200. 10.1007/BF03178082 9848052 PMC3453156

[36] Muthiah MN, Gias C, Chen FK, Zhong J, McClelland Z, Sallo FB, et al Cone photoreceptor definition on adaptive optics retinal imaging. Br J Ophthalmol. 2014;98(8):1073–9. 10.1136/bjophthalmol-2013-304615 24729030 PMC4112439

[37] Thevenaz P, Unser M. User-friendly semiautomated assembly of accurate image mosaics in Microscopy. Microscopy Research and Technique. 2007; 70(2):135–146. 10.1002/jemt.20393 17133410

[38] Hariri AH, Zhang HY, Ho A, Francis P, Weleber RG, Birch DG, et al Quantification of ellipsoid zone changes in retinitis pigmentosa using en face spectral domain-optical coherence tomography. JAMA Ophthalmol. 2016; 134(6):628–35. 10.1001/jamaophthalmol.2016.0502 27031504 PMC5317200

[39] Issa PC, Finger RP, Holz FG, Scholl HPN. Multimodal imaging including spectral domain OCT and confocal near infrared reflectance for characterization of outer retinal pathology in pseudoxanthoma elasticum. Invest Ophthalmol Vis Sci. 2009;50(12):5913–8. 10.1167/iovs.09-3541 19553619

[40] Gliem M, Zaeytijd J De, Finger RP, Holz FG, Leroy BP, Charbel Issa P. An update on the ocular phenotype in patients with pseudoxanthoma elasticum. Front Genet. 2013;4:4–14. 10.3389/fgene.2013.00014 23577018 PMC3617449

[41] Klien BA. Angloid streaks; a clinical and histopathologic study. Am J Ophthalmol. 1947;30(8):955–68. 20256332

[42] Affortit AS, Lazrak Z, Leze RH, Basdekidou C, Caputo G, Vignal C. En face spectral domain optical coherence tomography in a case of bilateral acute macular neuroretinopathy. Retina. 2015;35(5):1049–50. 10.1097/IAE.0000000000000362 25402220

[43] Itoh Y, Vasanji A, Ehlers JP. Volumetric ellipsoid zone mapping for enhanced visualisation of outer retinal integrity with optical coherence tomography. Br J Ophthalmol. 2016;100(3):295–299. 10.1136/bjophthalmol-2015-307105 26201354 PMC4936524

[44] Toto L, Carpineto P, Parodi MB, Di Antonio L, Mastropasqua A, Mastropasqua L. Spectral domain optical coherence tomography and in vivo confocal microscopy imaging of a case of Bietti’s crystalline dystrophy. Clin Exp Optom. 2013; 96(1):39–45. 10.1111/j.1444-0938.2012.00784.x 22908902

[45] Mansour AM, Uwaydat SH, Chan CC. Long-term follow-up in Bietti crystalline dystrophy. Eur J Ophthalmol. 2007;17(4):680–2. 17671952 10.1177/112067210701700434 PMC2507722

[46] Shi F, Chen X, Zhao H, Zhu W, Xiang D, et al Automated 3-D retinal layer segmentation of macular optical coherence tomography images with serous pigment epithelial detachments. IEEE Trans Med Imaging. 2015: 34(2):441–52. 10.1109/TMI.2014.2359980 25265605 PMC13698588

[47] Chen H, Chen X, Qiu Z, Xiang D, Chen W, et al Quantitative analysis of retinal layers' optical intensities on 3D optical coherence tomography for central retinal artery occlusion. Sci Rep. 2015; 5:9269 10.1038/srep09269 25784298 PMC4363859

[48] Vermeer KA, Mo J, Weda JJA, Lemij HG, de Boer JF. Depth-resolved model-based reconstruction of attenuation coefficients in optical coherence tomography. Biomed Opt Express. 2013;5(1):322–37. 10.1364/BOE.5.000322 24466497 PMC3891343

